# Dual-roles and beyond: values, ethics, and practices in forensic mental health decision-making

**DOI:** 10.1007/s11019-024-10247-2

**Published:** 2025-01-25

**Authors:** Sven H. Pedersen, Susanna Radovic, Thomas Nilsson, Lena Eriksson

**Affiliations:** 1 Centre for Ethics, Law and Mental Health, University of Gothenburg, Gothenburg, Sweden; 2 Forensic Psychiatric Clinic, Sahlgrenska University Hospital, Gothenburg, Sweden; 3 Department of Philosophy, Linguistics and Theory of Science, University of Gothenburg, Gothenburg, Sweden; 4 Institute of Neuroscience and Physiology, University of Gothenburg, Gothenburg, Sweden

**Keywords:** Forensic psychiatry, Empirical ethics, Pragmatism, Values, Dual-role dilemma

## Abstract

Forensic mental health services (FMHS) involve restricting certain individual rights to uphold or promote other ethical values – the restriction of liberty in various forms is justified with reference to health and safety of the individual and the community. The tension that arises from this has been construed as a hallmark of the practice and an ever-present quandary for practitioners. Stating this ethical dilemma upfront is a common point of departure for many texts discussing FMHS. But do we run the risk of missing something important if setting the ethical scene rather than exploring it? This paper draws on interviews with three types of interested parties in mental health law proceedings – patients, psychiatrists and public defenders, and seeks to tease out what values are enacted when they describe and discuss experiences of FMHS and court proceedings. In doing so, we find emphasized values such as acceptance, telling it like it is, atonement, normality, and ensuring the future. We find that well-delineated and separate values are not necessarily the basis for decisions. We also find potential for explanation and guidance in bringing ethical discourse closer to everyday practice.

## Introduction

Forensic mental health services (FMHS) encompass a multitude of different assemblies of laws, norms, care- and institutional practices in various parts of the world. Despite differences between jurisdictions, FMHS share key features and challenges in that they purport to manage and treat individuals who suffer from serious mental illness and who also pose a risk to the safety of others (Crocker et al. 2017). These fundamental similarities allow us to meaningfully discuss FMHS ethics as something that, at least partially, applies across jurisdictions despite the idiosyncrasies of FMHS legislation in different countries. In this paper, we use qualitative interview data to expand on the prevailing understanding of FMHS ethics and offer the suggestion that a practicable FMHS ethic may require a new and contextualized framing.

### Dual-roles and the framing of FMHS ethics

An important feature of FMHS ethics is the duality of caring for an individual while also using constraint and coercion to protect the community. It is presumed that decisions in the FMHS setting involve balancing these values in different ways and that a practicable ethic of FMHS would involve a resolution of how to balance them. This framing has prompted discussions about the dilemmas faced by professionals operating at the intersection between the justice system and psychiatry. The challenge of reconciling professional obligations to care for the individual and to serve the wider community has been discussed in relation to psychiatrists offering expert advice in court (Richardson and Machin 2000), clinicians in FMHS (Adshead and Sarkar 2005), and patients’ legal counsels (Abisch 1995).

In a review of the literature, Robertson and Walter (2008) discuss the *dual-role dilemma* which emerges when a psychiatric professional is bound by duties to more than one stakeholder or objective. They identify several different manifestations but note that the dilemma has typically been associated with forensic psychiatric practice, referring to the oft-cited papers of Stone (2008) and Appelbaum (1990, 1997). In his 1984 paper, Stone (republished 2008) questioned whether the dual commitment required of the forensic psychiatric expert witness can be justified, as it takes psychiatrists out of the ethical boundaries of their own field – which is thorny and contradictory enough as it is – and moves into the realm of justice. Stone believed that it could not, one of the reasons for this being that a doctor’s ethical duty is to alleviate suffering in individual patients, not ensure the well-being of unidentified masses. In response to this reasoning, Appelbaum (1990) argued that forensic psychiatry should be understood as an altogether different arena and role precisely *because* forensic psychiatrists operate outside the realm of a medical framework, where the ethical principles are weighted differently. Subsequently, Appelbaum concluded that forensic psychiatry is a different arena, in need of a specific ethical framework. Arguing against a framework of “mixed duties” (Appelbaum 1997, p. 243), he emphasized justice as the primary value guiding forensic psychiatrists.

Radden (2002) makes a case for general psychiatry needing a partly non-medical ethic, in recognition of the social and medical practices that distinguish the field from other bio-medical endeavours. She argues that psychiatry at its core is a normative project that seeks to reform or improve patients’ character, which together with other unique features calls for a distinctive professional ethic. The point is not merely that mental health care settings can give rise to specific ethical challenges – such as those related to involuntary treatment – but that psychiatric practice inherently operates within a distinct ethical terrain. Similar to how Appelbaum addressed the ethical responsibilities of psychiatric expert witnesses, Radden argues that an ethic grounded solely in medical principles is insufficient for psychiatry. While both Appelbaum and Radden discuss issues relevant to FMHS as a clinical practice, neither directly engages with FMHS itself.

### Empirical contributions

Bioethics have struggled to translate abstract theory into clinical practice (ten Have and Gordijn 2024). Alongside ambitions to find novel ways to develop normative theory, such challenges have contributed to the so-called “empirical turn” in medical ethics (Davies et al. 2015; Hedgecoe 2004; Kon 2009). In regard to involuntary psychiatric care, there are several examples of empirical studies investigating values and ethical reasoning, with a focus on what different actors describe as important values in the context of such care. Seeking to combine philosophical and social scientific inquiry in new ways (Dunn et al. 2012), Dunn and colleagues (2016) investigated the use of Community Treatment Orders (CTO) to enable situated analyses of how ethical principles could and should be translated in real-world medical decision-making. Drawing on interviews with psychiatrists, patients, and family carers, they conclude that there is no valid in-principle justification for CTO’s, but instead, the merits of CTO’s must be argued based on specific and concrete trade-offs on a case-by-case basis.

Valenti and colleagues (2014) explored the question of which values are important to patients in involuntary treatment, analysing interviews with patients involuntarily admitted to acute wards across England. Patients in the study described an array of practices that indicated a lack of respect on the part of staff and that limited patients’ freedom. Patients also recognized that admission to involuntary treatment had some benefits in the form of risk reduction for themselves as well as others. Identifying as patient values freedom, safety, and respect, and as staff values life and health, the study recommends using moral deliberation theory as a tool to address ethical issues and manage conflicts.

In a study investigating attitudes and experiences of informal coercion among mental health professionals, Valenti and colleagues (2015) concluded that professionals who often disapprove of such practices in theory still use them. Noting that coercion is ‘a moralised construct’ (Hoge et al. 1997), Valenti and colleagues relay that professionals found informal coercion not only effective but that their role and objectives demanded it. El Alti and colleagues (2022) – lending some support to the practices described by Valenti (2015) – argue that emancipation is not always a viable objective in FMHS. They discuss person-centred practices and describe how objectives such as adherence, security, alliance, and even limited forms of patient agency can be achievable in FMHS, but also how constraints within the institution and patients’ capacities limit how cooperative the decision-making can be (El Alti et al. 2022).

### The Swedish setting

Forensic care is the result of a criminal sanction in Sweden (Penal Code, Swedish Code of Statutes (SCS) 1962:700). Offenders with serious mental illnesses are considered responsible for their crimes, capable of criminal intent, and are sentenced in criminal courts. The requirements for a forensic care sanction are that the offender suffers from a mental disorder of a kind or degree that corresponds to psychosis and that the offence could have rendered a prison sentence. Those sanctioned to forensic care are detained in dedicated FMHS clinics. The continuation of the care is tried in mental health law proceedings (MHLPs) every sixth months, after an application from the clinic’s chief psychiatrist or their representative^1^. MHLPs fall under the remit of administrative courts with a judge leading the proceedings and determining the case along with two lay judges. An independent psychiatrist is appointed to aid their assessment by providing neutral medical expertise. The patient is represented by a public counsel provided by the state (Compulsory Psychiatric Care Act, SCS 1991:1128; Forensic Mental Care Act, SCS 1991:1129).

With the responsibility to determine whether FMHS ought to continue for a patient, the court becomes the setting where the definitive decision is made about whether the interventions or control that the clinic provides are necessary for each patient. The MHLP setting is often described as an arena where values such as autonomy, health, and fairness are being explored and weighed against each other and where the clinical setting is often in focus (see also Sjöström 1997; Zetterberg et al. 2014). The need to protect the public, the patient’s need for care, the patient’s freedom, the patient’s belonging to the community, and more, are all potentially at stake in this setting. This makes MHLPs a particularly fertile setting to explore valuation in relation to FMHS.

### Summary and aims

In sum, the discussion on the dual-role dilemma presumes that decisions in the FMHS setting involve balancing the values of autonomy, health, and public safety in various ways. Empirical studies indicate that within this general value conflict, a wide range of specific value issues and dilemmas emerge. In this study we seek to expand on such work by examining descriptions of the decision-making process surrounding the continuation of FMHS, which takes place in MHLPs. We search for valuation beyond what is explicitly stated in interviews about FMHS ethics and try to elaborate on what values are in fact made important in everyday decision-making. This move reflects a more pragmatist understanding of values which places the focus on the act and process of valuation and directs attention to how values are enacted (Davies et al. 2015; Dussauge et al. 2015; Morrison 2019). In line with Dewey’s (1913, 1939) understanding of values as inextricably linked with their active articulation, we will analyse interviews with interested parties in the FMHS setting and what emerges as important to them, or to the institutions and other actors that they describe. We will focus on how valuation is made visible when informants describe how they pursue, defend, or even violate certain objectives. We explore if and how they appear to relate to each other and – importantly - whether they allow us to see something which will expand the perspective on what is or ought to be encompassed within the ethic of FMHS.

Our aim is to make an empirical contribution concerning valuation in FMHS, which also has implications for the framing of FMHS ethical discourse. Attending to patients’, psychiatrists’, and public counsels’ reasonings and descriptions of the process of deciding whether ongoing FMHS ought to continue, we examine values as enacted in the interviewees’ descriptions of goals, strategies, practices and effects.

## Methods

The analysis carried out in this study draws on interviews from two previously collected datasets that include patients, public counsels and applicant psychiatrists involved as parties in MHLPs concerning general and forensic involuntary mental health care in Sweden. 24 participants with experience from MHLPs and FMHS were interviewed – 6 public counsels, 13 patients, and 5 psychiatrists. The recruitment and interviewing of professionals and patients were separate, as patient interviews were added when two projects were joined (previous studies from these projects, with more detail on primary analyses include Pedersen et al. 2020; Radovic et al. 2020). The overall sampling strategy was a form of purposive expert sampling, whereby individuals with relevant knowledge, expertise, and experience of the mental health law proceedings were sought. For professional participants, an initial purposive sampling was followed by asking participants whether they knew someone who would be of interest to the project. The resulting sample consisted primarily of participants who were currently active in the administrative courts in two larger Swedish court districts.

Patient participants were recruited at a forensic mental health inpatient clinic and a corresponding outpatient service in Sweden. Importantly, patients were interviewed for the same reasons as the professional participants were selected: to draw on their first-hand experiences with forensic mental health services and administrative court hearings. Recruitment in this context was opportunistic and broad. All available patients were offered information and those who volunteered and met the inclusion criteria were interviewed. Inclusion criteria required that the individual had experience of at least one mental health law proceeding under the Forensic Mental Care Act (SCS 1991:1129) and was able to communicate in Swedish to the degree that an in-depth interview was possible. Additional requirements were that patients did not suffer from acute psychosis that would have interfered with their ability to participate in an interview, as well as with safety measures^2^.

### Analytic procedure

The overall approach of the original projects sought an iterative and inductive process with data collection and analyses proceeding in tandem for each of the respective data sets 3. A first few interviews were carried out and preliminary analyses conducted by researchers with different disciplinary backgrounds. The initial analyses were used to inform the subsequent interviews which were in turn used to elaborate on codes and themes which had been identified.

The analysis presented here draws on interviews with patients, public counsels, and treating psychiatrists, respectively, collected within the two joined projects. It focuses specifically on how values are articulated as the interviewees describe and discuss different practices. The choice of professional categories was based on their role of representing the interests of the patient in some respect. The interviews were analysed by the first author; segments of data reflecting valuation or value-related practices were extracted and coded. Codes were then grouped and repeated analytic sessions were held to construct themes based on the codes. All authors participated in the analytic sessions, discussing themes and interpretations of segments. The first and last authors compiled the analysis.

## Ethics

The collection of data from FMHS patients for research is sensitive and requires care. However, there is a dire need for research to support clinical FMHS practice, and the current status of poorly supported, involuntary care over long time needs to be taken into account. Our reasoning in support of low-risk research in the field has been elaborated on elsewhere (Munthe et al. 2010; Pedersen et al. 2021).

Ethical approval was provided for interviews with patients by the regional ethical review board of Gothenburg (dnr 247 − 15). Written informed consent was collected prior to interviews. The interviews with professional actors about their area of expertise did not pertain to sensitive data as defined by Article 9 of the EU’s General Data Protection Regulation (Regulation (EU) 2016/679 of the European Parliament and of the Council). Therefore, they do not fall under the Swedish Act concerning the ethical review of research involving humans (SCS 2003:460) requiring ethical approval. Verbal informed consent was collected prior to interviews.

## Results

### Acceptance - adjustment of behaviour and alignment of thinking

The appropriateness of patients’ behaviour and attitude is a central matter of concern that appears to guide practices as well as evaluations of whether the care is having the desired effect. Professionals seek to persuade patients to adhere to the institution’s instructions, in terms of behaviour but also of thinking. As part of a treatment goal patients can be encouraged to internalize compliance with the pharmaceutical regimen that they will need to stick with for the foreseeable future and to make them proponents rather than mere recipients of their medication.

You should be prepared to continue medicating for the future, you should be prepared to defend your medication for the future. So even if another doctor comes and suggests, ‘Shouldn’t we try to discontinue this or something like that?’ you should say, ‘No, I want to continue with the medication for the future. (Psychiatrist 5)

For patients to be present in the court hearings is valued by the psychiatrists and some public counsels, as it is deemed to be simultaneously encapsulating a positive attitude towards one’s care and demonstrating a concrete willingness to translate that openness into practice. In the quote below a psychiatrist explains that one of the reasons that they try to persuade patients to participate in the mental health law proceedings is that such attendance in itself is a measure of acceptance, which in turn is a prerequisite for eventual release.

It’s also interesting because it can also demonstrate how involved one is in one’s own care. I mean, the goal is still to try to reintegrate these individuals into society, to gain an understanding that one has an illness. That’s one of the significant aspects, that one can have an understanding that one may need medication for the rest of one’s life. So, this acceptance and realization of it, it’s a huge part of it.” (Psychiatrist 1)

While accepting medication and participating in particular meetings or proceedings are fairly concrete instantiations of adjustment, they may or may not reflect an inner, more wholesale alignment. FMHS patients can have odd or abnormal thoughts, which clinicians seek to change. Working against such thoughts or expressions can be viewed as a way of influencing patients’ thoughts and worldviews to be more aligned with that of the surrounding society. One potent way of reducing risk is to shift and shape patients’ perception of the world and thus, hopefully, future conduct. The public counsel quoted below discusses how psychiatrists aim for a controlled inner world among patients.

Their goal is to win the client over. Their long-term goal is to convince the person in question about their worldview and their perception about what is right or wrong, how they ought to live their lives and what choices they ought to make. (Public counsel 5)

To patients, this striving towards inner change can translate as an institutional expectation of unconditional acceptance of clinicians’ assessments and wishes. In the excerpt below, a patient is asked to what extent the content of the care provided is sometimes discussed with their public counsel, beyond the question of whether or not they think they should receive it at all.

Patient: Well, I don’t usually oppose the doctor, I know that it doesn’t make that much of a difference. So I usually just say, “Yes, sure, I accept it.” And then you don’t go deeper into it, in that sense. I’m not the one who wages fights unnecessarily.

Interviewer: Have you ever had the thought that you’d like to put up a fight here, and you’d like things to be different?

Patient: In the beginning of my time, it was probably like that, I really wanted to get out of there when I was [under the Forensic Mental Care Act]. But you eventually learn how it works, and you know what’s expected of you, and so after a while, the better you feel you take fewer fights because you already know everything that’s required. Like, you know what’s required to get out of there, and you know what the doctor wants, and so on. (Patient 8)

In this telling, there is a gradual recognition of a set of expectations that will need to be met by any patient hoping to be released. Such a realization, in parallel with feeling healthier, will make one less prone to objections as it would be counterproductive. Later in the interview, the same patient describes how disputing facts or issues presented in the MHLP would run counter to a presentation of self that is necessary to appear healthy and harmless.

But it’s important to submit to the doctor’s will. Because if you were to try to be on the same level [as the doctor] and keep saying “You’re wrong” all the time, you wouldn’t get anywhere. It’s important to paint a picture of yourself as being healthy and not dangerous. And fighting against it goes against the evidence. So you have to be very careful and calm and show that you’re rational and can accept decisions even if they are difficult. And say: “Yes, well, I guess I’ll have to wait another six months,” for example. (Patient 8)

Interviewees describe different practices designed to promote desired behaviours in patients. Among them are explicit interventions such as written conditions for furlough or denying privileges with reference to disagreement. Such explicit constraints on patients’ behaviours are used as tools to communicate to patients which actions or attitudes are desirable. The patient quoted below makes the case that denying furlough can be a punishment for non-compliance. In their understanding, non-compliance is equated with mental illness by staff.

As long as you disagree with your care team or with staff you’re never getting out anywhere. Because if you disagree, then they consider you ill. And if you do as they say, then you’re well. (Patient 17)

Institutional security procedures appear to foster certain behaviours or mindsets. Patients need to adjust to curfews and searches as part of everyday life. In the quote below, a patient describes how entering the premises after furlough and undergoing controls requires a change of mindset. They describe needing to shift from “freedom” to “restriction” and readying themselves for what the institution expects of them.

It was a huge ordeal every time to always reset from freedom to restriction and go through that, the same process there at [the clinic] when you go through that gate and they have to touch and check in your bag and rummage here and there… It’s very difficult. And then mentally reset from freedom to restriction, and everyday report there at 19:00, 19 sharp. (Patient 13)

It is the logic of the institution itself that is addressed, rather than actions carried out by any individual members of staff, when patients describe a state of acceptance necessary to navigate everyday realities of forensic psychiatric care. Acceptance presents as an important institutional value, not only of the care provided, but of the underlying reasons for why it is provided and why parts of it must continue. This is enacted through adjustments of behaviour and signs of alignment with the rationale for care and treatment, such as participation in activities at the clinic or going along with what is suggested.

### Telling it like (you think) it is

In contrast with, and perhaps as a reaction to the institutional value of acceptance, interviewees also describe strategies for protecting remaining freedoms. Forced medication, intrusive searches, restraints, and locked doors are described as external influences that infringe on the movement and boundaries of a patient’s body. Internal states are also impacted, as patients adapt to external pressures. The freedom to have emotions, goals, and subjective worldviews can be circumscribed by how professionals interpret or manage such internal processes. The patient below describes how they suppress their emotions and expressions with the goal to hide any signs of mental illness.

I would like to speak to the administrative court and tell them precisely those words I’ve said to you now, but I don’t dare to, I refuse, maybe they’ll be angry with me or upset, they’ll never let me out from there. (Patient 16)

For this patient, disguising their true assessment of the situation they find themselves in becomes a strategy to protect and increase chances of future release. In reaction to such circumstances, some professionals aim to support the patient’s thoughts and worldviews as a way of valuing a patient’s freedom to express themselves and to think freely. The public counsel quoted below describes having to work hard to make room for patients’ worldviews, in a context where those worldviews are treated as dangerous. The ‘they’ who get upset in the example are in this instance psychiatrists or members of the court who favour the pursuit of other values.

And then I say “No” and I go in emphatically, I reason, I argue, and I’m really clear. And then what can happen is… There are different reasons, but as I said, one of the reasons they get upset could be that they are worried that the client is reinforced in this incorrect worldview or this mistaken attitude in relation to how they see things. (Public counsel 5)

Patients describe how “telling it like (you think) it is” is not merely a matter of what you feel able or allowed to express, but the communicative structure within which you are expected or enabled to do so. A court proceeding’s speaking order can have an encouraging or dampening effect, depending on how it is exercised. Being left out of conversations about themselves until the tail end of the hearing can make it difficult for patients to contribute. The next quote illustrates such a practice of supporting a patient’s ability to express themselves. The patient is involved in each part of the conversation and finds themselves able to influence the discussion.

The judge asked my counsel questions, and I got to speak as well. Then they asked the doctor, and the doctor and I could talk to each other in front of the judges. The expert and I had a conversation, in front of the judges. And it has never happened before. Normally, you’re supposed to just sit there quietly. I mean, from what I’ve experienced before. Let only the counsel speak for themselves and let only the doctor talk. Then, if there’s something I want to say, I will do it at the end, and it’s not always then that you remember what they have said before. (Patient 7)

Patients describe different ways to carve out some form of freedom within the FMHS system. Some manage to claim space and make themselves heard. Others accept the external constraints but try to maintain command over their own thoughts or motivations. One interviewee described how they preserved some autonomy by sticking to their own, private reasons for seemingly unconditional compliance. Another interviewee deduced that the only way of getting out is to try to stay inside:

Forensic psychiatric care can be endless; it has no end. My theory is that I’ll reach the end of this by trying to stay here, and then I’ll be kicked out. (Patient 2)

### Atonement and assuming responsibility

For some interviewees, a key objective of FMHS is to atone in some way for the crimes committed. The crime is recognized as a moral wrongdoing that requires some form of rebalancing. Accepting guilt, subordination, or apologizing are key features in this rebalancing. Some consider FMHS to be a form of legitimate punishment that needs to be endured because of previous wrongdoings. This is reflected in one patient’s statement below, where they discuss how to act in relation to the care institution. Because of what they have done, they must submit to the will of the psychiatrist.

But I suppose it’s a bit like I said, I touched on before, that it’s a punishment after all, that you’re here for. So you’re the subordinate party, because you’ve put yourself in this position yourself. And if you understand that then you’re well on your way out of here. If you don’t then you have a long way to go. And I suppose that’s kind of how the doctor sees it too, I think. (Patient 8)

Admitting guilt and apologizing to the professionals helps the patient build trust. It can also ease some of the weight of guilt. The patient quoted below speaks of the relief they experienced after having admitted to their crime. They also describe a change in the relationship with psychiatrists and judges, who had previously been harsh but later became helpful and benevolent.

At first, I had denied and said that it wasn’t me. But then I realized that I had ruined someone’s life with my crime. And it was my fault. So, I confessed. After that, everything became easier. I gained more self-confidence, and I received more help. (Patient 11)

The examples above illustrate patients who submit to the authorities as a signal of their own moral betterment. In their description, atonement is a shift that happens in relation to professionals, or to society with professionals as representatives. Another way of seeking moral reparation is to attempt to restore the damage caused by the crime, such as repairing relationships with those harmed. When asked about agency and moral responsibility in the role of patient, the patient below describes how they sought forgiveness from and reparation with the victims.

I was given the opportunity. I got to go home and meet my family, they were the ones subjected to the crime, and repair the relationships. And I suppose that’s a way to take responsibility. But after I got treatment I quickly got better. And that made it possible to do that. If you’re seriously ill, chronically ill, then maybe that opportunity isn’t there. (Patient 15)

### Normality

Not being that different from others, or not being unnecessarily exposed as being different from others, comes through in the material as something important to safeguard and make possible. Interviewees speak of acting to support the normality or belongingness of patients in a variety of ways, as reflected in the subsections below.

#### Hiding the unseemly

It is sometimes patients’ *apparent* normality and equal standing that appears to be in focus. Interviewees describe presenting a respectable front or hiding abnormalities in ways that bring patients closer to the norm and make them more acceptable to others. The psychiatrists quoted below describe a habit of phrasing applications and statements made in court with a minimum of unflattering information included. The practice is intended to protect the patient in two ways: from painful memories, and from being shamed in front of the courtroom actors. Being too explicit about the patient’s history or functioning is understood as showing them up.

You try to write courteously and then you attend the hearing and then you say as little as possible, I think, because you don’t want to… I mean it’s no fun to hear all the misery that you’ve experienced and that. (Psychiatrist 5)

I usually try not to violate [sv: kränka] the patient more than necessary in a court proceeding. This means that I don’t need to address all the strange delusions a patient has if I feel the hearing is going my way. (Psychiatrist 4)

#### Explaining and contextualising the seemingly abnormal

Some patients, on the other hand, describe wanting their story to be told. Sharing information can help make their current state understandable and appear less abnormal. This practice is described in the quote below. The patient describes how aspects of their history within FMHS are no longer being discussed. Reflecting on how the care institution controls that information, they relate a previous experience of submitting a written statement to a court hearing as a way of explaining and contextualizing their symptoms and functioning.

Interviewer: What did you write?

Patient: That I grew up being sexually abused and began to run away from home because I didn’t want to live in the same house as my stepfather, because he was the one abusing me, and the whole story of my life then. (Patient 1)

In this understanding, understating or glossing over information risks being counterproductive as it is the painful and problematic that serve as explainers. That which would otherwise sort under the heading strange behaviour, can instead be categorized as understandable, if still problematic, reactions.

#### Presentation of self and personhood

The value of appearing normal and less troubled is also reflected in patients’ descriptions. In patients interviews this is linked to convincing others of one’s own capacities. Below, a patient describes how attending to their physical appearance before court hearings is a way of signalling capacity and readiness for release.

It looks better than coming with stains on your clothes or something like that. Because, I mean, you should be able to live a life outside the walls too. (Patient 1)

Some public counsels highlight a practice that seems to strive for another form of normalcy. They describe becoming sought after as legal representatives by acting as though the patient is like everyone else; showing acceptance and respect for the patient as they are. Having coffee with a patient or talking about their interests are examples brought forth as a particular strategy when dealing with FMHS clients.

I think I’m pretty straight with my patients and respect their integrity and can talk about their interests and that. What are their interests and what seems important to them, what level I go in at. I mean, adjust your approach and speak the customer’s language [sv: Tala till bönder på bönders vis]. (Public counsel 1)

I quickly became very popular with the patients, because they had never met anyone who came home and sat down to drink coffee. (Public counsel 3)

The counsels are describing how seemingly mundane interactions such as showing interest or accepting a cup of coffee inject an unusual and appreciated sense of personhood in a context where roles tend to be restricted to ‘patient’ and ‘professional’.

In a care setting, the equivalent kind of personhood would require recognizing and acting on moments of distress with the compassion of a fellow human. The patient quoted below describes trying to help a fellow patient manage their distress when staff would not. While soothing and hugging may not help the patient with their mental illness, it is nonetheless considered an appropriate and normal response to overt distress.

I mean, at the previous place I was at, there was a guy […] who had significant problems with psychoses and paranoid thoughts and such. But, I mean, I helped him. I was there to hug him and talk to him. Tried to make him feel ’it’s okay,’ but the thing is, it’s like talking to a wall. He says ’okay, you understand,’ and then, five minutes later, it starts all over again. So, I had to tell the staff there, ’What are you guys doing? You need to help him. It’s not my job to help him. I’m a patient here.’ But I helped him more than they did, you know. (Patient 5)

The practices described above represent ways of approximating versions of normality that serve different purposes: to protect from shame or pain; to explain the otherwise anomalous; to demonstrate capacity, or to offer recognition of a shared humanity. While seemingly disparate, they nevertheless enact an overarching strive towards that which unites rather than sets apart.

### Ensuring the future

Ensuring the future by way of handling mental illness and preventing violence is described by psychiatrists and public counsels as predominant practices and core objectives of FMHS. The stakes as portrayed in the material could not be higher; mental illness is described as deadly, and treatment or control of it as saving lives or preventing serious harm. There are different strategies for ensuring the future: to extend and ideally make permanent supporting structures (durability), and to pre-empt potential future serious events by identifying and swiftly acting on presumed pre-cursors (pre-emption). These are presented in separate subsections below.

#### Durability/continuity

The psychiatrist below discusses how the organization of care incentivizes short-sighted decision-making, describing how maintaining neuroleptic treatment is essential to prevent mental health from deteriorating.

My basic attitude is really that you should never reduce medication unless you have reviewed the medical records. Because, I mean, you don’t know what the limit is when people get sick. Then you can ruin people’s lives, some people have gotten sick one time too many. I mean, if they have recovered after the first time, reasonably healthy after the second time, after the third time, maybe long-lasting? (Psychiatrist 5)

Learning to live in the community is a challenge for some patients and durable functioning within the norms and demands of society is enacted as a value through calling for multi-modal treatment and skills training. None of the informants describe this kind of rehabilitation taking place, but are instead calling for it, requesting specific interventions or processes. The patient quoted below, having talked about treatments that they had asked for from their care team, describes how a holistic treatment programme would help them manage life.

Because if you’ve become so ill at some point that you end up there, I believe you need lifelong support. Regardless of whether you are free or locked up. It must be the intention that when you are locked up, the rehabilitation begins there. With all the resources that can be provided. And then you need both medication and psychological help for strategies. And to try them out and maybe try medications, try strategies. Learn to live again! (Patient 3)

Durability/continuity partly overlaps with Acceptance, as the latter is often presented as a prerequisite for, or means to, bringing about a desirable future defined by the twin absences of pathology and violence.

The wide scope that follows from ensuring the future makes efforts to ensure that the patient receives FMHS care legible as means to promote health. These can come in the form of active commitment decisions, or in the form of refraining from actions that could have prevented FMHS care. In the quote below, a public counsel indicates that they intentionally hold back in court, with an eye to the well-being of the patient.

The role of the advocate is quite challenging, I think, because you have to make that assessment. If I sit and strongly question… Many times, to be honest, it’s obvious that this patient needs to continue to receive care. He is a danger to himself, he can’t manage on his own, or something like that. And to just strongly be the patient’s megaphone and strongly question the medical foundations, I don’t think that’s what one should do. (Public counsel 6)

#### Pre-emption

A public defender explains below how practices that relate to minor events in the present can be understood as valuing the future well-being of some unknown person. They talk of criminal courts and the psychiatric assessments made before sentencing when describing how certain minor events can be taken as indicative of serious future harm. Timely intervention in the form of involuntary commitment can thus be understood as a key practice in preventing serious harm.

So you think like this that this harassment is really just a prelude. Next time it’ll be an illegal threat, then it’ll be assault, and then we have murder. (Public counsel 3)

The treatment of psychiatric symptoms, particularly using injectable anti-psychotic drugs, is described as a way of preventing violence; mental illness is understood as causally connected to violent behaviour. The psychiatrist below describes how either neuroleptics or commitment are key to reducing the risk posed by the prototypical FMHS patient.

If it’s a patient sanctioned for a violent crime who starts using drugs or who refuses to take their medication, then you actually tend to commit them immediately. (Psychiatrist 4)

## Discussion

In our analysis, *acceptance* comes through as a value so central to FMHS that one might describe it as a constitutive part of an institutional logic. Patients who are able to accept their subordination and align their self-perception, worldview, and behavior with the expectations of the care institution are more likely to be seen as having met the objectives of care. The *durability* of such adjustment and alignment is a key objective for (FMHS) professionals, who aim to foster recognition and acceptance of illness and inherent vulnerability, ultimately leading to lifelong dependence on a medical regimen.

The pronounced institutional emphasis on these values appears to curtail patients *telling it like (they think) it is*, as expression of disagreement or displeasure can in and of itself be interpreted as a sign of ill health, regardless of the content or nature of the disagreement. This can also be discerned in the tension arising when legal professionals attempt to defend their clients´ right to speak the truth as they see it, which to care professionals can appear as downright counterproductive and undermining the long-term pursuit of durable conformity.

Acceptance comes in two renditions in the material; an in-principle version that moves the patient over time towards health through insight, and an in-practice version that both promotes and results in strict obedience. It is worth noting that practices associated with the latter version could be construed as promoting third party safety over patient health, thus an instantiation of the dual-role dilemma. This understanding requires us to assume that something lies behind obedience as a truer objective. If we allow obedience to be a potential objective, we can perhaps gain new perspectives on important findings such as the pervasiveness of non-participation in FMHS (Söderberg et al. 2020, 2022, 2023) or how staff can be simultaneously uncomfortable with and still use informal coercion in involuntary care (Valenti et al. 2015). Non-participation in this light – as a form of alignment – would not be an unwanted side effect, contrary to policy (e.g. Patient Act, SCS 2014:821), but indeed an objective in its own right. Similarly, informal coercion could be expected if adjustment is a central objective.

The value of *normality* is enacted in several and at times conflicting ways. Psychiatrists may attempt to avoid detailed descriptions of symptoms or abnormal behaviours to spare the patient distress, thus enacting normality by hiding the abnormal. Seeking normality in this way can conflict with patients’ attempts to make the seemingly abnormal understandable by providing explanatory context. Such a drive towards wanting to highlight rather than hide can also be tied to telling it like it is. They both involve having your perspective and interpretations taken seriously and both values are difficult to realize in an institution that is structured around professional actors rather than patients owning the narrative (relating to the epistemic status of the patient, see Fricker 2011). Being seen and met as an ordinary person to have a chat with over a coffee, or whose overt distress generates concern and comfort, constitutes an additional, noteworthy, and coveted form of normality.

The emphasis on *ensuring the future* serves as a backdrop against which to understand the institution’s focus on achieving acceptance and normality. With a task that is forward-looking and concerned with more than the immediate future, FMHS are required to identify early signs of a detrimental development. However, finding precise, early signs of specific outcomes is difficult (Solmi et al. 2023), and a cautious clinician might interpret any sign of deviance from the expected course as a sign of either mental deterioration or increased risk for violence, or both. Allowing distant outcomes to weigh against the present skews the comparison in favour of caution; the relatively minor liberty of expressing one’s viewpoints here and now is balanced against the potential risks of future events, such as suicide or murder. Consequently, ensuring a “safe” future would necessitate controlling any deviance at an early stage, allowing only highly restricted and muted behavioural expressions, as reflected in our results.

The focus on normality echoes the research by Gildberg and colleagues who noted that “reconstructing normality” (2012 p.103) seemed to be the primary, immediate objective of FMHS inpatient care. Yet there is also an aspect of hiding abnormality in our results, that cannot be explained by reference to making the patient be more normal or be treated as normal. One reading of our results is that concealing abnormality may be seen as an act of respect for the patient – avoiding unnecessary discussions about their flaws to prevent disrespect. However, some patients reject this interpretation. If we instead consider the concealment of abnormality as an enacted value in its own right, reminiscent of what Chappell and Jeppsson (2023) call psychiatry’s normality bias, it can help broaden the discussions on how stigma associated with mental illness or criminal histories might be addressed (West et al. 2014) or the potential role of FMHS in re-institutionalizing individuals who are marginalized and excluded from society (Turner 2004). Programs devised to ameliorate stigma by explaining the evolution of mental illness may, for example, not satisfy those who value downplaying signs of mental illness over contextualizing mental illness.

It is noteworthy that the concept of *atonement* seems to align patients’ and professionals’ valuation. Displaying guilt and a commitment to repair is a direct and open way of enacting something valuable for patients, which is also valued by professionals. There is however a risk of insincere atonement on the part of the patient, or distrust in patients’ remorse from professionals. It is instrumentally rational for professionals to be cautious and try to pre-empt serious risks, as we have noted above. It is also instrumentally rational for patients to project an image of normalcy and acceptance^4^, even if it means hiding some of their core selves (as discussed in Marklund et al. 2020), as it may bring them closer to being deemed ready for discharge. In this, the care risks becoming based on the appearance of collaboration. If atonement is implicitly sought by professionals and valued by patients, then collaboration may be better served by making it explicit and enabling discussions about *how* it can be sought.

## Limitations

The analysis is based on interviews with patients and professionals who describe the practices of themselves and others. Access to observations of behaviours in some form would likely make visible other forms of valuation and expand the analysis. Similarly, there are many roles within FMHS which are not represented among the informants of this study and interviews with representatives of other roles are likely to provide additional information. Observational studies within FMHS, interviews with other actors, and analyses of clinical documentation would be avenues to pursue in future research.

Much like the ethical discourse concerning professional duties – such as the dual-role dilemma – our analysis places the focus on individual practitioners, thereby overlooking the fact that both practitioners and patients operate within a very specific and regimented environment. In our study we stop short of analysing how valuation can be enacted in material settings, but we see hints in testimonies about, for example, the impact of entering the premises or people having their purses searched. These indicators echo the work of Lewis and Holm (2023) and Pols (2006), who explore the material aspects of seemingly immaterial values. Further research that considers the material and procedural aspects of valuation would complement this study and contribute to the development of a more practically useful theory.

## Conclusion

This study draws on empirical material to further a discussion on how ethics may be conceived of in the very particular setting that FMHS presents. This empirical and pragmatist approach accesses a register that is closer to FHMS daily practice and opens up the discussion about possible ways of understanding the purpose and rationale of FMHS. Our findings resonate with writers (e.g. Clouser and Gert 1990; Hedgecoe 2004; Kohlen 2009; Pols 2015) who argue that a principle-based ethics such as that espoused by Beauchamps and Childress (2001), and institutionalized in law and ethics committees, are insufficient and may run the risk of obscuring rather than throwing light on day-to-day ethical conundrums. Two aspects seen in our study seem particularly relevant in the context of FMHS:

Firstly, our study finds that well-delineated and separate values need not be the basis for decisions. The rational balancing of autonomy, health, and security in clinical practice is hindered by challenges such as whether it is even possible to differentiate these values in what is observable in the clinic. While this is not to say that such a balancing is ethically unjustified or unhelpful, it seems insufficient as guidance if clinicians’ decisions need to account for values closer at hand. If, for example, practitioners are striving to achieve acceptance in their clinical encounters, it may be more useful to clarify which forms of acceptance are desirable, rather than categorizing acceptance as a practical manifestation of the values inherent in the dual-role dilemma. If normality is front and centre, perhaps clinicians and patients can reach a truer dialogue if it is made clear how each of them understands what it is to normalize something abnormal – whether it is more helpful to make the seemingly abnormal understandable in context or to hide it from view, and for whom the hiding is done. This has implications for care planning and the patient’s progression through care. It is also a foundation for partnership between patient and professionals to share an understanding of the task at hand (Sahlsten et al., 2008).

Secondly, our results indicate the relevance of discussing an ethic for FMHS which goes beyond the dual-role dilemma. Aspects of what has been described as psychiatry’s normative project (Radden 2002) and normality bias (Chappell and Jeppsson 2023) are evident, but there are other possible ways of framing it. For example, the putative role of FMHS in re-institutionalization (Turner 2004) may involve shielding the community from unwanted persons. If this is the case, it must be reflected in our ethical discourse. Importantly, discussing a partly non-medical ethical framing is not a rejection of the dual-role dilemma or its relevance to FMHS ethics. Incorporating descriptions of valuating practices helps bring to the fore challenges and objectives which stem from other influences than those prescribed in law or policy, but which nevertheless must be considered by clinicians. Thus, in combination with the dual-role framework and conceptual models for core objectives in FMHS (e.g. Hartvigsson et al., forthcoming), our results can help clinicians disentangle objectives which are not found in policies, but which are nonetheless unavoidable.

While our analysis is distinctly local, some themes seem likely to be found across jurisdictions. Stigma and re-institutionalization – to the extent that it is happening – are global phenomena (cf. Kaliski, 2013) and forensic patients’ lack of control over their circumstances has been described in a number of jurisdictions (e.g. Askola et al., 2018; Barnao et al., 2015; Jacob 2012). However, we argue that local research is essential to ensure that the results are closely aligned with practice. In addition to addressing the limitations noted in the previous section, we recommend conducting similar studies in other jurisdictions to help bridge the divide between ethical theory and practice in the specific contexts where it will be applied.

## Footnotes

1 Under Swedish law, a sentence of forensic care can be with or without Special Discharge Review (SDR), depending on the estimated risk for serious recidivism. For those with SDR, administrative courts hold the decision to continue or end compulsory care. For those without SDR, the court is only tasked with trying whether care may continue. Approximately 80% of patients are sanctioned with an SDR (Swedish National Forensic Psychiatric Register, RättspsyK, 2023).

2 Of those individuals who volunteered to participate, all met the inclusion criteria. Most likely, those who would not have met these criteria were never reached by the information, either due to the lack of information in other languages, or due to them being isolated from other patients in the hospital, or due to intermediate staff choosing not to approach them.

3 This approach is inspired by Grounded Theory as a set of principles and practices rather than prescriptions (Charmaz 2006). While the use of previously collected data prevents the application of certain elements, we adhere to the view (e.g. Braun and Clarke 2006) that thematic analysis is in itself a valid analytic approach. The analysis employed in this paper draws on both of the traditions noted above.

4 Atonement could be read as a version of acceptance: Acceptance is enacted in relation to perceived expectations from the institution be it in terms of behaviour (adjustment) or outlook (alignment), whereas atonement is specifically directed towards patients’ stance on events ‘on the outside’ that preceded or caused the stay in FMHS. While it is the case that tellings of atonement are often accompanied by descriptions of how articulating one’s own responsibility/guilt resulted in smoother and more positive interactions with caregivers as well as courts, it also contains an element of personal relief that is important in its own right.
